# Editorial: Diagnostic and predictive roles of computational cardiovascular hemodynamics in the management of cardiovascular diseases

**DOI:** 10.3389/fbioe.2026.1804586

**Published:** 2026-02-18

**Authors:** Fuyou Liang, Yonghui Qiao, David Perpetuini, Harvey Ho

**Affiliations:** 1 Department of Engineering Mechanics, School of Ocean and Civil Engineering, Shanghai Jiao Tong University, Shanghai, China; 2 Key Laboratory of Hydrodynamics (MOE), School of Ocean and Civil Engineering, Shanghai Jiao Tong University, Shanghai, China; 3 State Key Laboratory of Ocean Engineering, Shanghai Jiao Tong University, Shanghai, China; 4 School of Power and Energy, Northwestern Polytechnical University, Xi’an, China; 5 Department of Engineering and Geology, University G. d’Annunzio of Chieti-Pescara, Pescara, Italy; 6 Auckland Bioengineering Institute, The University of Auckland, Auckland, New Zealand

**Keywords:** cardiovascular diseases, computational methods, hemodynamic, model personalization, multi-physics model

## Introduction

Current clinical management of cardiovascular diseases relies heavily on medical imaging. However, structural indices derived from medical images, such as the degree of stenosis or aneurysm diameter, may not fully reflect the functional severity of disease (Liu Y. et al., 2025). Computational hemodynamics, therefore, serves as a critical complementary modality, offering functional insights that fill this gap (Makropoulos et al., 2024). This Research Topic presents 16 studies that collectively demonstrate computational cardiovascular hemodynamics not merely as a theoretical method for basic research, but as an evolving clinical tool capable of complementing diagnosis, predicting progression, and optimizing intervention.

## Quantitative analysis of hemodynamic parameters for precise diagnosis

A primary limitation in clinical diagnosis arises from the reliance on invasive procedures or subjective assessments (Zhang et al., 2023). Computational hemodynamics addresses this by providing non-invasive functional quantifications (Li et al., 2025). Wang et al. tackled the limitations of using invasive pressure wire to assess the hemodynamic impact of intracranial atherosclerotic stenosis. Their study demonstrated the concordance between angiography-derived quantitative flow ratio and invasive non-hyperemic pressure ratio, establishing a wire-free methodology for assessing functional ischemia.

To address the challenge of interpreting complex imaging data, Guo et al. developed an echocardiographic video-driven multi-task learning model (IE-CAD) that simultaneously estimates the Gensini score and cardiac functional parameters (e.g., global longitudinal strain, left ventricular ejection fraction, global work efficiency). This approach automates the diagnosis and grading of coronary artery disease, thereby effectively overcoming the subjectivity inherent in manual visual assessment. Additionally, Fan et al. proposed a multidisciplinary framework combining numerical simulation with deep learning to enhance the hemodynamic analysis of aneurysms, demonstrating how artificial intelligence can accelerate complex diagnostic workflows. Collectively, these studies highlight a paradigm shift towards non-invasive, automated, and functionally integrated diagnostic protocols that reduce reliance on operator subjectivity and invasive instrumentation.

## Hemodynamic metrics as biomarkers of risk or progression of disease

Risk stratification relying exclusively on geometric metrics often lacks the ability to fully capture the heterogeneity of disease (Hesse et al., 2024). To address that issue, hemodynamic metrics predicted by computational models, such as wall shear stress (WSS) and its derivatives, may serve as additional biomarkers, yet their predictive accuracy is contingent upon high-fidelity biomechanical representation (Qiao et al., 2022). Goetz et al. conducted comprehensive fluid-structure interaction (FSI) analyses on 101 intracranial aneurysms (AnXplore), revealing that traditional rigid-wall assumptions overestimate WSS and induce significant deviations in the prediction of oscillatory shear index. Consequently, incorporating vascular wall compliance appears to be important for the precise characterization of rupture risk metrics.

Extending the pursuit of realism from passive compliance to active biological modulation, Qin et al. employed a multimodal approach involving ultrasound, histology, and wire myography in a rat model to demonstrate that a decline in arterial smooth muscle active contractile force correlates with inflammatory exacerbation and matrix degradation. Yue et al. utilized FSI analysis with a multi-layered anisotropic model to explore the biomechanical function of the intraluminal thrombus (ILT) in abdominal aortic aneurysms, finding that the ILT provides a significant “cushioning effect” that attenuates wall stress and mitigates stress concentrations caused by medial degradation.

In addition to these methodological refinements, recent studies have validated hemodynamic metrics as sensitive biomarkers across diverse pathologies. Armour et al. demonstrated a direct correlation between wall shear stress (WSS) patterns and disease severity in pulmonary arterial hypertension, thereby substantiating the utility of flow metrics in tracking disease progression. Similarly, Chen et al. addressed the clinical discrepancy between anatomical significance and functional risk, revealing that even “mild” coronary stenosis induces flow disturbances critical to plaque progression. Furthermore, Yu et al. elucidated the compression mechanics inherent to myocardial bridging, while Lv et al. quantified cerebral blood flow regulation defects in patients with an incomplete circle of Willis. Taken together, these findings indicate that computational modeling offers a quantitative framework to evaluate the hemodynamic impact of complex anatomical features and mechanical properties, thereby better assessing the dysfunctional severity or predict the progression of cardiovascular disease.

## Computational hemodynamics as a tool for optimizing clinical interventions

The final phase of clinical management focuses on precise intervention and long-term prognosis, where computational modeling provide a robust platform for pre-operation planning, device optimization, and post-operation surveillance (Luan et al., 2023; Liu T. et al., 2025). Šeman et al. reviewed the utility of computational modeling in managing valvular heart disease, highlighting its capacity to bridge the gap between clinical observation and theoretical prediction in complex pathologies like mixed valvular disease. By forecasting post-procedural hemodynamic profiles, these models can serve as a useful tool for reducing diagnostic uncertainty.

Building upon its predictive capability, device-specific modeling is essential for optimizing interventional procedure. Yin et al. developed a fully coupled FSI model for bioprosthetic aortic valves and elucidated that kinematic parameters, especially wall shear stress and leaflet deformation undetectable by standard imaging, may provide critical insights for identifying the mechanical precursors of structural deterioration. Yang et al. demonstrated that the Niagara catheter’s novel helical flow inducer significantly enhances perfusion efficiency. Through computational fluid dynamics, they confirmed that the induced helical flow suppresses stagnant zones, thereby mitigating thrombogenic risk.

Effective clinical management extends beyond the immediate success of intervention, necessitating post-procedural assessment to monitor functional recovery and hemodynamic stability. Lv et al. highlighted the importance of integrating lifestyle into post-stenting care by investigating the combined effects of stent design and patient exercise intensity. Their findings revealed that exercise-induced hemodynamic variations significantly alter wall shear stress profiles, suggesting that physical activity levels can be prescribed to minimize restenosis risk. Paparella et al. utilized 2D speckle-tracking echocardiography (2D-STE) to quantify cardiac mechanics following AF ablation, identifying functional recovery and reverse remodeling that standard volumetric assessments fail to capture. Jensen et al. systematically quantified hemodynamics after aortic coarctation repair, demonstrating that despite anatomical correction, abnormal flow patterns often persist.

## Conclusion

The studies compiled in this Research Topic highlight the potential clinical significance of shifting from static structural assessment to dynamic functional evaluation in cardiovascular medicine. By validating non-invasive diagnostic tools, elucidating biomechanical risk factors, optimizing interventional devices and predicting long-term postoperative outcomes, computational hemodynamics is demonstrating its value as an indispensable component of precise patient management.
